# Mobile Media Content Exposure and Toddlers’ Responses to Attention Prompts and Behavioral Requests

**DOI:** 10.1001/jamanetworkopen.2024.18492

**Published:** 2024-07-10

**Authors:** Sara Jane Webb, Waylon Howard, Michelle Garrison, Sarah Corrigan, Shayeleen Quinata, Lani Taylor, Dimitri A. Christakis

**Affiliations:** 1 Center on Child Health, Behavior and Development, Seattle Children’s Research Institute Seattle, Washington; 2 Psychiatry and Behavioral Sciences, University of Washington, Seattle; 3 Department of Public Health, Purdue University, West Lafayette, Indiana; 4 Pediatrics, University of Washington, Seattle, Washington; 5 Special Olympics International, Washington, DC

## Abstract

**Importance:**

With personalized touch-screen tablets, young children can choose content and engage in play-like activities. However, tablets may also reduce shared engagement as the action of viewing or touching the screen is often not visible to nearby adults. This may impact communicative gazing and pointing, which is critical to the formation of shared awareness and in turn supports language development.

**Objective:**

To assess the association of tablet media content with toddlers’ responses to joint attention prompts and behavioral requests.

**Design, Setting, and Participants:**

This cohort study took place at a behavioral research laboratory and included toddlers who were aged 18 to 32 months with neurotypical development who were recruited from a volunteer and community sample. Toddlers engaged with a real toy or 3 different types of tablet content (ie, viewing video of toy play, playing with a digital toy, or playing a commercial game) while an experimenter delivered joint attention prompts. Data were acquired from June 2021 November XX 2022, and data analysis occurred from January 2023 to May 2024.

**Main Outcomes and Measures:**

Main outcomes included child response to joint attention (number of prompts with joint attention response per number of prompts delivered) and child response to behavioral request (ie, the prompt on which the child responded to the behavioral request). Measures included crossed random effects, Wald tests, and likelihood ratio tests.

**Results:**

In this study, 63 toddlers were enrolled, and data from 62 were included (31 female [49%]; mean [SD] age, 26.1 [3.4] months; median [IQR] age, 25.0 [18.6-32.6] months). When toddlers were playing a commercial game on a tablet, they responded to fewer joint attention prompts (crossed random effects model, −0.15; 95% CI, −0.24 to −0.06 prompts) and male toddlers took longer to acknowledge a behavioral request (interaction of content and sex, −0.75; 95% CI, −1.36 to −0.17). The negative impact of the tablet game was larger as child age increased (τ = −2.30; 95% CI, −0.05 to 0; *P* = *.*03). Greater media use at home was associated with decreased responding to joint attention prompts during the tablet game (*ρ* = −0.47; *P* < .001), while better language skills were associated with more joint attention during play with a real toy (*ρ* = 0.31; *P* = .02).

**Conclusions and Relevance:**

In this cohort study, a touch-screen tablet game was associated with decreased joint attention among toddlers and they were less likely to respond to a behavioral request. In a laboratory setting, it was difficult for toddlers to engage in social-communicative interactions with adults when using a tablet media device.

## Key Points

**Question:**

Are different types of tablet media content associated with toddlers’ responses to joint attention prompts and behavioral requests?

**Findings:**

In this cohort study of 63 toddlers, participants responded to fewer joint attention prompts and took longer to acknowledge a behavioral request when playing with a tablet commercial game.

**Meaning:**

These findings suggest that touch-screen, engaging, tablet games may inhibit early social-communicative interactions.

## Teaser

This cohort study examines the association of screen time with responses to joint attention prompts and behavioral requests among toddlers with neurotypical development.

## Introduction

In the last 20 years, the format of media use has significantly changed from centralized TV use to personalized handheld tablets and smartphones.^1,2,3,4^ The American Academy of Pediatrics that discourages all screen time before 18 months and recommends parental involvement from 18 to 24 months.^5^ With tablets, young children can direct the action, including viewing content, playing with 2 dimensional toys, or playing games. However, the shift to tablets raises several questions as individual usage may diminish opportunities for dyadic interactions, which are critical for social, cognitive, linguistic, and emotional development.

There is a growing body of research about the impact of tablet use on child behavior. Playing with digital toys on a tablet, compared with traditional physical toys and print books, reduces infant-parent reciprocity^6^ and toddler-infant verbal interactions.^7^ Furthermore, toddlers learn less from videos with touch-screen features than in real-life interactions.^8^ Independent use may lead to further attention narrowing, decreased responsiveness to external cues, and missed opportunities for social reciprocity.^9^

Observational studies have linked excessive media use generally with attention problems, language delay, and social-emotional concerns.^10,11,12^ A recent meta-analysis^13^ and a well-controlled cohort study^14^ found that early screen use was associated with developmental and language delays, but the mechanism remains elusive. Additionally, executive functioning is diminished in toddlers who have screen time that exceed guideline recommendations,^15^ and preschoolers with high touch screen use (ie, more than 30 min per day) have more inhibitory control issues 12 months later.^16^ While beneficial outcomes have been proposed for education and sustained attention, these are often tested at a low-dose compared with actual media use patterns.^17^

In a proof-of-concept study, we hypothesized that the mechanism mediating the association between increased tablet media use and decreased language outcomes is due to disruption of joint attention. Joint attention is a critical learning mechanism during toddlerhood (ie, age 12 to 36 months), with increased consistency and efficiency of responses.^18^ Shared awareness supports language development,^19,20,21^ and joint attention is associated with language 1.5 to 6 years later.^22,23^ Specifically, we propose that highly engaging touch-screen commercial apps compared with other content types inhibits responding to joint attention and behavioral requests, and reduced joint attention during tablet use will be associated with lower language ability.

## Methods

This cohort study followed the Strengthening the Reporting of Observational Studies in Epidemiology (STROBE) reporting guideline. Written informed consent was obtained from a parent and human participants regulatory oversight was provided by Seattle Children’s institutional review board.

### Protocol

We conducted a 1 time point, laboratory-based, cohort study of 63 toddlers that included an experimental measure of joint attention during tablet media use, parent report of child media use and motor skills, and an observational measure of language ability. Data were acquired from March 2021 to September 2022. Analyses were conducted from January 2023 to May 2024.

### Participants

In this study, 129 participants were recruited using community e-flyers and a local university-based participant pool. To be eligible for the study, toddlers had to be healthy, to have been born full-term, to have had a normal birth weight, to have had exposure to English, and to have at least attempted to use a mobile touch-screen device; 78 toddlers met the inclusion criteria. Toddlers were also screened to have no parental concerns of developmental delays (eg, language delay, autism spectrum disorder). Parents reported child race and ethnicity using the National Institutes of Health criteria. Race categories included American Indian or Alaska Native, Asian, Black or African American, Native Hawaiian or Other Pacific Islander, White, more than 1, other, and not reported, and ethnicity categories included Hispanic or Latino, not Hispanic or Latino, or not reported. Sixty-three toddler-parent dyads attended the in-person laboratory visit.

### Child Characteristics

A parent report, developmental screening tool, the Ages and Stages Questionnaire-3 (ASQ), was used to assess communication, gross motor, fine motor, problem solving, and personal-social domains. The Preschool Language Scale-5 (PLS) was used to assess expressive language; data were missing from 1 participant due to fussiness.

### Media Use

The primary caregiver reported media use (of any type) for the week before the visit. Data were missing for 3 participants.

### Media Joint Attention Task

The media task was a 30-minute within-participant crossover experimental task with 5 randomly sequenced activities. The toddler participant, caregiver, and experimenter were seated at a trapezoid table with the toddler in a booster chair between the experimenter and parent. All media were presented on a 10-inch iPad with a child-protected case, lying flat on the table. One child did not provide usable data on the media task.

Conditions included (1) object toy (a farm toy); (2) tablet watching toy (ie, passive viewing of a noncommercial video of a child playing with the farm toy); (3) tablet toy (ie, a app in which the child places the missing puzzle piece in a farm themed image); (4) tablet game (an app game in which the child cares for farm animals, such as sheering sheep); and (5) a favorite app identified by the caregiver (15 [24%] reported having a favorite app and Fruit Ninja was used for those without a favorite app). We do not include this condition further. Tablet conditions were presented in guided access mode, which did not allow the child to exit the app.

Within each condition, the experimenter placed the toy or tablet in front of the toddler and said “Let’s play!” In phase A the toddler interacted with the materials for 1 minute, and the caregiver and experimenter did not engage the child unless child initiated. In phase B, every 30 seconds, the experimenter delivered a joint attention prompt (2 × name + point, 2 × name + look)^24^ toward 1 of 8 targets (eg, wall posters and toys) located in the room for 2 minutes and concurrent with the child’s interaction with the materials. In phase C, the caregiver engaged with the child in a naturalistic way for 2 minutes, and the experimenter did not initiate any interactions. In phase D, the experimenter concludes the interaction by asking for the toddler to give the item back to them (“Give it to me”) with hand gestures^24^; up to 3 prompts were used. If the toddler refused to comply, the experimenter asked the caregiver to remove the item. At the end of each condition, the experimenter switched materials for the next condition; this took approximately 10 seconds (range, 5 to 30 seconds).

### Joint Attention Coding

Child response to joint attention (RJA) was coded live from phase B as the total number of successful joint attention responses (looking to the experimenter and then following the prompt to the target location) divided by the number of prompts, providing a percentage score. A higher value reflects more successful joint attention.

Child RBR was coded live from phase D as the prompt for which the child responded to the behavioral request. We did not require the child to comply but instead acknowledged that the request was made (eg, saying no was coded as a pass). A 3 reflects response on the first ask, 2 as response on the second ask, 1 as response on the third ask, and 0 as noncompliance. Data from 10% of participants were double coded, with reliability more than 90% of all measures.

### Statistical Analysis

We first examined the association of the media task conditions with RJA and response to behavioral request (RBR). A crossed random-effects model^25^ was used to account for variability across participants and media conditions in RJA and RBR across 4 tasks. Analyses were generated using the lme4 version 1.1-31 (R Project for Statistical Computing). For analyses, the reference value for format was the object toy contrasted to the 3 tablet conditions. The reference value for content was the less engaging conditions (ie, object toy, watching tablet toy, and tablet toy) with a contrast for the tablet game. Child age was centered at 25 months, female was the reference group for sex with a contrast for male. Wald tests were used to evaluate the significance of fixed effects and likelihood ratio tests were used to evaluate random effects. We also evaluated information criteria (Akaike information criterion [AIC]) between models with the same fixed effects. Effect size was evaluated via pseudo *R^2^* values for the proportion reduction in each variance component, as well as with total *R^2^*, the squared correlation between the actual RJA or RBR outcomes and the RJA or RBR outcomes accounted for by the fixed effects. Because any potential biases and loss of power due to missing data were likely benign (2.8% missing values on the media task), we proceeded with the maximum likelihood estimate estimator rather than fitting multiple imputation or full information maximum likelihood based models. Second, we used Spearman ρ to examine the association between the RJA responses across the 4 conditions, home media use, and child language and motor skills. All statistical analyses were conducted with a significance level of α = .05, and all tests were 2-tailed unless otherwise specified.

## Results

### Participants

Of the 63 toddlers who were included in the study, 31 (49.2%) were female, the mean (SD) age was 26.1 (3.4) months, and the median (IQR) age was 25.0 (18.6-32.6) months. The parent-identified race included 1 American Indian or Alaska Native toddler (1.6%), 3 Asian toddlers (4.8%), 57 White toddlers (90.4%), and 3 toddlers (5.8%) did not have race reported. Five toddlers (7.9%) were Hispanic or Latino and 55 (87.3%) were non-Hispanic. Most primary caregivers had a college education or greater (51 [81.0%]) and 45 (71.4%) worked full-time or part-time.

### Baseline Characteristics

Although none of the parents had concerns about their child’s motor ability, 8 (12.7%) and 13 (21.0%) of the toddlers had ASQ values on the gross motor and fine motor above the cut point for concerns, respectively. The Preschool Language Scale-5 (PLS) was used to assess expressive language; all scores were in the average to above average range (mean [SD], 106.1 [11.5]). Toddlers had a mean (SD) of 280.8 (361.3) minutes of media per week; 17 (28.3%) had 70 minutes or less per week; while 12 (16.0%) had 420 or more minutes per week. Age was not associated with amount of media use.

### RJA During the Media Task

As seen in Table 1, the toddlers had fewer mean (SD) RJA responses during the tablet game condition (object: 0.59 [0.32]; tablet watching toy: 0.71 [0.33]; tablet toy: 0.65 [0.34]; table game: 0.50 [0.34]). Three models without factors (ie, empty means models) were estimated to partition the total RJA variation. Relative to a model specifying a single residual variance, there was significant variability in mean RJA across participants (likelihood ratio tests, 6.12; *P* = *.*01), and across conditions (likelihood ratio tests, 11.66; *P* < *.*001), such that 17.9% of the RJA variation was due to mean differences across participants, 7.6% was due to mean differences across media conditions, and the remaining 74.6% was due to the participant by condition interaction (ie, residual variance). Random effects CIs indicated that 95% of the individual participant and task RJA means were expected between 0.63 and 1.03 (mean, 0.83), and 0.70 and 0.96 (mean, 0.83), respectively.^26^ That is, while there was somewhat greater variation between participants than between tasks, both sources of variation were significant, and thus their random effects were retained.

**Table 1.** Toddler Performance by Media Content

Media content	RJA		RBR Prompt, No. (%)^a^			
	Mean (SD)	Median^b^				
			First	Second	Third	Fail
Object	0.59 (0.32)	0.50	35 (56)	9 (14)	3 (5)	16 (25)
Tablet watching toy	0.71 (0.33)	0.75	52 (82)	6 (10)	2 (3)	3 (5)
Tablet toy	0.65 (0.34)	0.75	45 (71)	3 (5)	2 (3)	13 (21)
Tablet game	0.50 (0.34)	0.50	40 (63)	3 (5)	2 (3)	18 (29)

Abbreviations: RBR, response to behavioral request; RJA, response to joint attention.

^a^ N = 63.

^b^ RJA range is 0.0 to 1.0.

Sequential models (Table 2) were then tested to examine task factors (ie, format, content) and participant factors (ie, age, sex) of RJA. The fixed intercept for the estimated mean RJA was 0.84 (95% CI, 0.77-0.92). Format and participant factors of sex and age were not significant.

**Table 2.** Sequential Models and Task and Participant Factors Associated With RJA

Coefficient	Parameter estimate (SE)			
	Model 2	Model 3	Model 4	Model 6
Intercept (γ_000_)	0.83 (0.02)	0.83 (0.04)	0.84 (0.04)	0.84 (0.04)
Age (γ_001_)	NA	NA	−0.00 (0.01)	0.01 (0.01)
Sex (γ_002_)	NA	NA	−0.04 (0.04)	−0.06 (0.06)
Content (γ_010_)	NA	NA	−0.15 (0.03)	0.17 (0.06)
Format (γ_020_)	NA	NA	0.06 (0.03)	0.04 (0.05)
Age × sex (γ_003_)	NA	NA	NA	−0.01 (0.01)
Age × content (γ_012_)	NA	NA	NA	−0.03 (0.01)
Age × format (γ_022_)	NA	NA	NA	−0.01 (0.01)
Sex × content (γ_011_)	NA	NA	NA	0.01 (0.08)
Sex × format (γ_021_)	NA	NA	NA	0.03 (0.06)
e_tis_	NA	0.05	0.04	0.04
U*_00s_*	NA	0.01 _ID_	0.01 _ID_	0.01 _ID_
U*_0i0_*	NA	0.01 _task_	0 _task_	0 _task_
U*_01s_*	NA	NA	NA	0.03 _ID (Content)_
Participants, No.	NA	62	62	62
Tasks, No.	NA	4	4	4
Observations	NA	245	245	245
Marginal *R^2^*	NA	0	0.07	0.10
Conditional *R^2^*	NA	0.26	0.26	0.44

Abbreviations: γ_000_, grand mean intercept; e_tis_, residual error term for each trial, item, and participant; ID, personal identification number; NA, not applicable; RJA, response to joint attention; SE, standard error of parameter estimates; U*_00s_*, random effect associated with participants; U*_0i0_*, random effect associated with item i.

**Figure 1.**
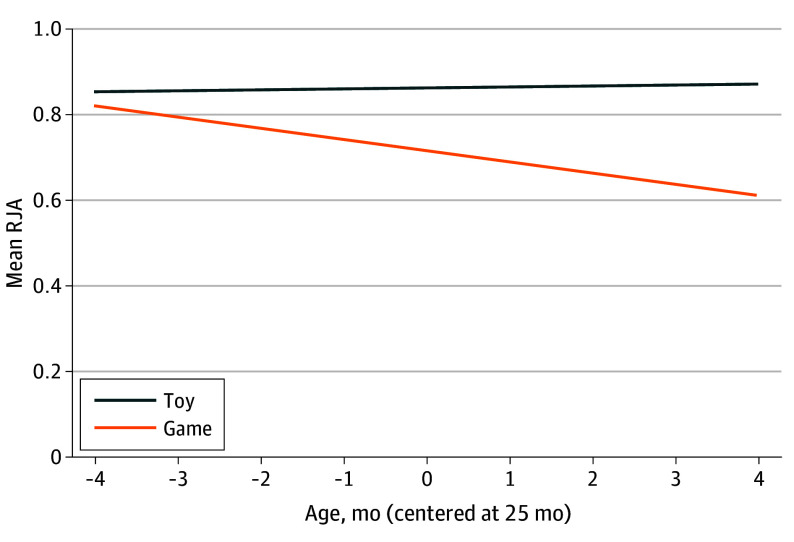
Response to Joint Attention (RJA) by Content Across the Distribution of Child Age (Centered at 25 Months) Simple slopes with confidence regions.

The simple main effect size of content was significant (−0.15; 95% CI, −0.24 to −0.06), such that the tablet game was associated with lower RJA (Cohen *f^2^* = 0.06). This task factor accounted for 99.2% of the task variation with no significant task intercept variation remaining, (likelihood ratio tests,  0.001; *P* = *.*98). Results indicate the factor content varied significantly over participants (likelihood ratio tests, 11.61; *P* = *.*003). An overall model *R^2^* was calculated as the square of the correlation between the RJA estimated by the fixed effects and the actual RJA for each trial (*R^2^* = 0.07; 95% CI, 0.02 to 0.14, for the model fixed effects). Interaction terms to assess moderation of the factor content by child age and sex were then added (*R^2^* = 0.10; 95% CI, 0.04 to 0.18). Results indicate a significant child age by content interaction (−0.02; 95% CI, −0.05 to 0), which reduced the residual variance by 6.4%, and the random content slope variance by 17.2%; however, significant participant random content slope variation remained (likelihood ratio tests, 9.76; *P* = *.*008). Child age was not associated with RJA within the object toy conditions. However, this factor was significantly associated with RJA within the tablet game condition. Every 1 month increase in child age was associated with a −0.03 (95% CI, −0.05 to 0) decrease in RJA (*τ* = −2.30; 95% CI, −0.05 to 0; *P* = *.*03) (Figure 1).

### RBR During the Media Task

Earlier successful RBRs were more likely to be seen in the tablet watching condition than the other conditions (object: 16 fails [25%]; tablet watching toy: 3 [5%]; tablet toy: 13 [21%]; tablet game: 18 [29%]) (Table 1). Relative to a model specifying a single residual variance, there was significant variability in mean RBR across participants (likelihood ratio tests, 29.49; *P* < *.*001) and across conditions (likelihood ratio tests, 11.97; *P* < *.*001), such that 33.8% of the RBR variation was due to mean differences across participants, 6.4% was due to mean differences across conditions, and the remaining 59.9% was due to residual variance. Random effects CIs indicated that 95% of the individual participant and task RBR means were expected to fall between 0.89 and 3.64 (mean, 2.26) or 1.67 and 2.86 (mean, 2.26), respectively. Therefore, there was relatively more variability across participants than across tasks (Table 3).

**Table 3.** Participant and Task Factors and RBR

Coefficient	Parameter estimate (SE)			
	Model 2	Model 3	Model 4	Model 6
Intercept (γ_000_)	2.26 (0.11)	2.26 (0.19)	1.99 (0.28)	1.93 (0.30)
Age (γ_001_)	NA	NA	−0.03 (0.03)	−0.08 (0.06)
Sex (γ_002_)	NA	NA	−0.02 (0.22)	0.06 (0.30)
Content (γ_010_)	NA	NA	−0.50 (0.29)	−0.19 (0.32)
Format (γ_020_)	NA	NA	0.53 (0.29)	0.46 (0.32)
Age × sex (γ_003_)	NA	NA	NA	0.05 (0.07)
Age × content (γ_012_)	NA	NA	NA	−0.02 (0.05)
Age × format (γ_022_)	NA	NA	NA	0.05 (0.05)
Sex × content (γ_011_)	NA	NA	NA	−0.75 (0.30)
Sex × format (γ_021_)	NA	NA	NA	0.02 (0.03)
e_tis_	0.96	0.87	0.87	0.86
U*_00s_*	0.47 _ID_	0.49 _ID_	0.51 _ID_	0.52 _ID_
U*_0i0_*	NA	0.10 _task_	0.04 _task_	0.04 _task_
Participants, No.	62	62	62	62
Tasks, No.	NA	4	4	4
Observations	239	239	239	239
Marginal *R^2^*	0	0	0.05	0.07
Conditional *R^2^*	0.33	0.40	0.42	0.44

Abbreviations: γ_000_, grand mean intercept; e_tis_, residual error term for each trial, item, and participant; ID, personal identification number; NA, not applicable; RBR, response to behavioral request; SE, standard error of parameter estimates; U00s, random effect associated with participants; U0i0, random effect associated with item *i*.

A series of 2-way interactions of task and participant variables were added to the model to examine the extent to which task (ie, format or content) and participant variables (ie, age or sex) moderate RBR (Table 3). The addition of these 6 two-way interactions resulted in an overall model (*R^2^* = 0.08; 95% CI, 0.02 to 0.15), which reduced the residual variance by 1.3%. The fixed intercept for the estimated mean RBR was 1.93 (95% CI, 1.42 to 2.54). The simple main effect size was not significant for age and sex. There was a nonsignificant simple main factor format (0.46; 95% CI, −0.18 to 1.07) and content (−0.12; 95% CI, −0.70 to 0.47). However, there was a significant sex by content interaction (−0.75; 95% CI, −1.36 to −0.17), which reduced the residual variance by 1.3%.

**Figure 2.**
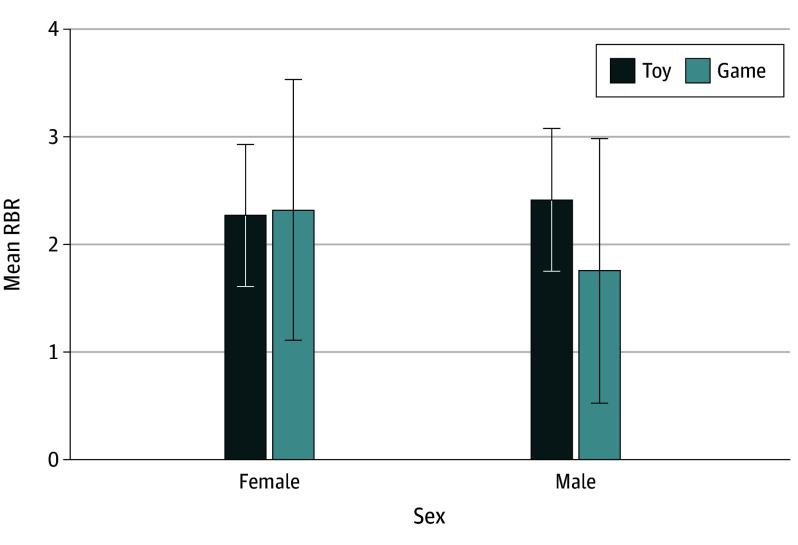
Sex by Media Content Interaction on Model Associated With RBR A higher score reflects earlier acknowledgment of the request for returning the tablet across the toy conditions (average of object toy, tablet watching toy, tablet toy) compared with the tablet game. RBR indicates response to behavioral request.

Next, the model was simplified. Relative to a model with all possible 2-way interactions (AIC = 759.27), a reduced model (AIC = 740.17) with only the significant sex by content interaction (and main effect size) did not have significantly worse fit (likelihood ratio tests, 9.10; *P* = .77); the simpler model was retained. Results indicate a significant sex by content interaction (−0.69; 95% CI, −1.27 to −0.15), where relative to the content factor for females (0.04; 95% CI, −0.71 to 0.87), the content factor for males was less positive by −0.69. During the tablet game, male toddlers had less willingness to acknowledge the requests to relinquish the tablet compared with other conditions (Figure 2)

### RJA, Media Use, and Language

Greater media use at home was associated with decreased RJA during the tablet toy (ρ = −0.42; *P* = .001) and tablet game (ρ = −0.47; *P* < .001) conditions. Additionally, a higher expressive language standard score was associated with more joint attention during object toy play (ρ = 0.31; *P* = .02) and better fine motor skills were associated with more joint attention during the tablet game (ρ = 0.31; *P* = .01). There were no significant associations with RBR.

## Discussion

In this cohort study, playing with a highly engaging tablet game was associated with fewer responses to joint attention prompts among male and female toddlers and less acknowledgment of a behavioral request among male toddlers. The association of the tablet game with RJA became larger as age increased. This suggests that tablet commercial games may be detrimental to early social-communicative interactions, particularly if they are reducing or replacing real toy play, parent-child dyadic activities, or peer play.^27^ Our RBR fail rate was between 20% to 29% for the tablet conditions and is consistent with Munzer et al,^28^ who demonstrated that 25% of 30-month old children exhibited tantrum behavior after removal of a tablet even after a short exposure.

### Commercial Games vs Toy Play

Play with physical objects is often driven by both endogenous attention (the child’s capacity to self-direct attention) and exogenous attention (the salient features of the displayed activity). Real toys also allow for multidirectional motor coordination, cognition, and symbolic play; all of which influence language acquisition.^29,30^ In contrast, other studies have proposed that touch-screen experiences are primarily influenced by exogenous attention^31,32^ and constrain the use of symbolic options. For example, apps reduce the number of allowable movement inputs and actions are spatially and temporally constrained. The real-world consequences a shifting from self-drive exploration to constrained play remains to be established.

### Caveats

Our results are derived from a laboratory task, allowing us to standardize the media, length of exposure before the prompts, and limit the complexity of the surrounding environment. We use prompts from a well-validated joint attention task that has shown significant associations with language development.^24,33,34^ During the task, the toddlers had limited areas of potential attention salience—the toy or tablet, the other adults, and the 8 room objects. Thus, we were able to isolate the association of the media content with child communicative responses.

Additionally, toddlers had difficulty responding to joint attention prompts and behavioral requests for real toys and the tablet game. However, joint attention to real objects was positively associated with language; thus, toddlers may recognize that real toys afford opportunities for dyadic play and learning. Additional focus on child initiations and parent interaction during natural situations will assist in understanding whether or not decreased joint attention per se is associated with decreased language or if it is the interaction with the specific type of content.

### Limitations

This study has limitations. This study was conducted from March 2021 to September 2022, which occurred during the end of the COVID-19 pandemic and is a preliminary, proof-of-concept study. Caregivers’ sensitivity to pediatric guidelines and self-increased use of media during the COVID-19 pandemic may have affected not only who decided to participate but reported media use.^35^ Thus, more direct assessments of media use (eg, app trackers) and assessment in home environments with more naturalistic probes are needed to fully understand real-life use patterns. Our sample was also limited in racial and ethnic diversity.

## Conclusions

In this cohort study of neurotypical toddlers, a highly engaging commercial game for the tablet and marketed for toddlers decreased responses to joint attention bids and decreased behavioral responses. This suggests that even in a laboratory setting, toddlers are less likely to engage with proximal attentive adults when using a tablet media device. This may diminish important opportunities for learning and social emotional development.

## Notes

Supplement.
Data Sharing Statement
